# Effects of clopidogrel, prasugrel and ticagrelor on prevention of stent thrombosis in patients underwent percutaneous coronary intervention: A network meta‐analysis

**DOI:** 10.1002/clc.23536

**Published:** 2021-03-11

**Authors:** Wenwen Chen, Chen Zhang, Jian Zhao, Xiuxiu Xu, Heqin Dang, Qiang Xiao, Yuanmin Li, Haifeng Hou

**Affiliations:** ^1^ Department of Cardiology, the Second Affiliated Hospital Shandong First Medical University & Shandong Academy of Medical Sciences Tai'an China; ^2^ School of Public Health and Management Binzhou Medical University Yantai China; ^3^ Department of Epidemiology Shandong First Medical University & Shandong Academy of Medical Sciences Tai'an China

**Keywords:** clopidogrel, network meta‐analysis, percutaneous coronary intervention, prasugrel, stent thrombosis, ticagrelor

## Abstract

**Background:**

Clopidogrel, prasugrel and ticagrelor, acting on platelet P2Y12 receptor, are commonly used for prevention of stent thrombosis (ST) among patients who underwent percutaneous coronary intervention (PCI). This study aimed to compare the effects of these drugs by a systematic review and network meta‐analysis.

**Hypothesis:**

Efficacies of clopidogrel, prasugrel and ticagrelor on preventing ST are not the same.

**Methods:**

PubMed, Embase and Cochrane Library were searched for randomized controlled trials (RCTs) that investigated the effect of clopidogrel, prasugrel, or ticagrelor on prevention of ST in patients who underwent PCI. The efficacies between groups were compared by a Bayesian network meta‐analysis, by which the pooled odds ratios (ORs) and 95% confidence intervals (CIs) was calculated.

**Results:**

Fourteen studies and 46 983 participants were included in this study. The pooled results illustrated that clopidogrel, prasugrel and ticagrelor were effective on prevention of ST. Patients treated with prasugrel (OR = 0.30, 95% CI = 0.052 ~ 0.73, *P* < 0.05) and ticagrelor (OR = 0.25, 95% CI = 0.035 ~ 0.65, *P* < 0.05) had lower incidence of ST compared to those treated with clopidogrel. Patients treated with ticagrelor showed similar frequency with those in prasugrel group (OR = 0.86, 95% CI = 0.22 ~ 2.3, *P* > 0.05). No significant heterogeneity was observed across included studies.

**Conclusions:**

Our findings suggest that prasugrel and ticagrelor are more effective than clopidogrel on prevention of ST among patients underwent PCI. Simultaneously, there is no significant difference in the prevention of ST between prasugrel and ticagrelor.

## 1 INTRODUCTION

The approach of percutaneous coronary intervention (PCI) with intracoronary stents has been widely used to prevent the occurrence of myocardial infarction (MI) in patients with acute coronary syndrome (ACS).^1^, ^2^ However, it is noteworthy that stent thrombosis (ST) is one of the severe complications following PCI, potentially leading to death.^3^ Results from the ADAPT‐DES study indicates that early ST (within 30 days) attributes to about 40% of mortalities among individuals who undergo PCI. During 2 years follow‐up, all‐cause mortality in these patients is significantly higher than those without MI, ST or clinically relevant bleeding (27.2% vs. 2.7%).^4^ Therefore, it is increasingly concerned to reduce the incidence of ST after PCI treatment.

As is known, the occurrence of ST is associated with patient‐, lesion‐, procedure‐, and post‐procedure‐related risk factors.^5^ Platelets play an important role in the pathophysiology of ST. Implantation of stent struts initiates platelet activation and adhesion, followed by thrombus formation, which results in early ST.^5^ Therefore, inhibition of platelet function is considered effective for preventing the occurrence of ST. Currently, one of the clinical approaches for inhibition of platelet aggregation is dual antiplatelet therapy (DAPT), which is based on the combination of aspirin with one of P2Y12 inhibitors (i.e., clopidogrel, prasugrel and ticagrelor).^6^ Studies have focused on the differences in the efficacies of P2Y12 inhibitors. Pharmacodynamic studies have suggested that the effect of clopidogrel is weaker than prasugrel or ticagrelor.^7^ Clinical trials and real‐world studies discovered that patients using clopidogrel suffered from higher incidence of ST compared to those treated with prasugrel or ticagrelor.^8^, ^9^, ^10^, ^11^ However, up to date, the difference in the incidences of ST between prasugrel and ticagrelor has not been clearly elucidated. Two studies reported that prasugrel was more effective than ticagrelor for preventing ST^12^, ^13^; however, other studies released insignificant results.^14^, ^15^, ^16^ Here, we conducted a systematic review and network meta‐analysis to compare the efficacies of three P2Y12 inhibitors (i.e., clopidogrel, prasugrel and ticagrelor).

## 2 METHODS

### 2.1 Search strategy

PubMed, Embase and Cochrane Library were searched for randomized controlled trials (RCTs) that compared the effect of clopidogrel, prasugrel, and/or ticagrelor in patients who underwent PCI. The search terms were “ticagrelor or brilinta”, “clopidogrel or plavix”, “prasugrel or effient”, and “ST”. These databases were searched for studies published up to January 12, 2020. RCTs registered in www.clinicaltrials.gov and major international cardiology meetings (American College of Cardiology (ACC), American Heart Association (AHA), and European Society of Cardiology (ESC)) were also identified. Inclusion criteria were: (a) RCTs, (b) comparison of clopidogrel, prasugrel or ticagrelor, (c) patients underwent PCI, (d) patients with follow‐up at least 6 months.

The primary end‐point was the rate of ST individually (as per the Academic Research Consortium definition).

### 2.2 Data extraction and quality assessment

The following data were collected from eligible studies: first author name, year of publication, region, type of study, duration of follow‐up, number of participants, gender of the subjects, and treatment regimens. The data were independently collected by two authors. The discrepancies were resolved by discussion with a third author.

Quality of included studies was assessed in accordance with the Cochrane Risk of Bias Tool, which contains seven domains: (a) random sequence generation, (b) allocation concealment, (c) blinding of participants and personnel, (d) blinding of outcome assessment, (e) incomplete outcome data, (f) selective reporting, and (g) other bias. Two authors completed quality assessment separately, and any discrepancies would be resolved by a third author.

### 2.3 Statistical analysis

We performed statistical analyses with Stata Version 14.0 (Stata Corp, College Station, TX, USA) and R Version 3.4.2 (R Development Core Team, Vienna, Austria). We carried out a Bayesian network meta‐analysis using the “gemtc” package for R. We calculated odds ratios (ORs) and 95% confidence intervals (CIs) for comparisons between groups. We analyzed the convergence of Markov chain Monte Carlo chains for all model parameters using trace plots and Gelman‐Rubin diagnostic statistics. With regard to heterogeneity test, a *P* < 0.10 indicated statistically significant. The potential publication bias was estimated by funnel plot asymmetry.

## 3 RESULTS

### 3.1 Characteristics of included studies

**FIGURE 1:**
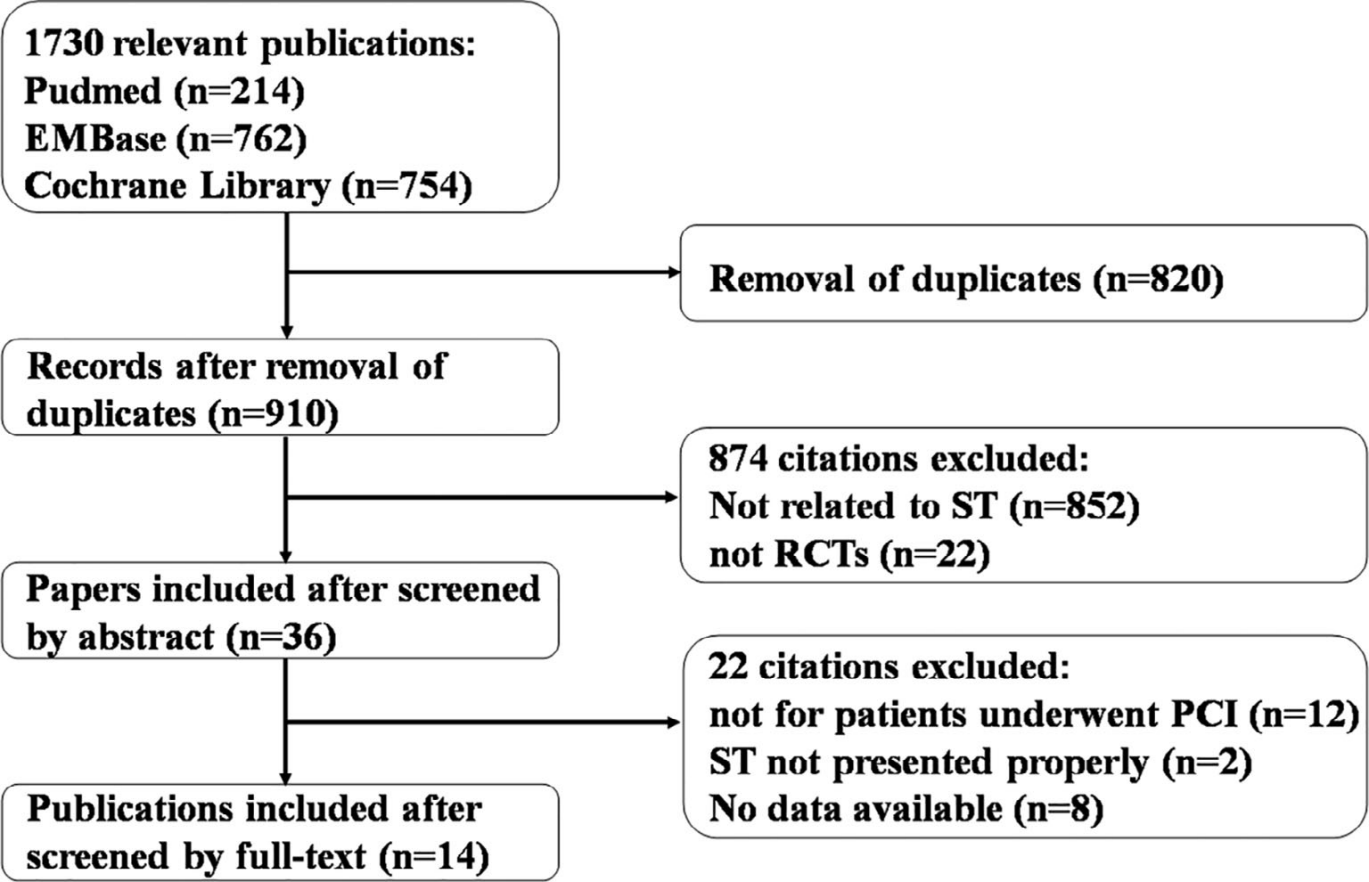
Flowchart of search strategy and article selection

Our literature search returned 1730 studies. After elimination of duplicate results, 910 studies were reviewed. After exclusion of 874 studies by title and/or abstract, 36 articles were reviewed in full‐text. Finally, 14 studies^8^, ^14^, ^15^, ^16^, ^17^, ^18^, ^19^, ^20^, ^21^, ^22^, ^23^, ^24^, ^25^, ^26^ that met all criteria were included in our meta‐analysis (Figure 1), with 46 983 participants. The duration of follow‐up was 11.1 ± 2.9 months, ranging from 6 to 15 months. As shown in Table 1, the majority of included studies were based in Asia, and the minority was from the America, Australia, Europe and other regions. Most studies included more males than females. Eight of 14 studies were multicenter studies. Six studies compared the difference of ST incidences between clopidogrel and ticagrelor, while four studies compared the difference between clopidogrel and prasugrel, and three studies compared ticagrelor with prasugrel. Only one study compared the difference between three agents.

**TABLE 1:** Study characteristics of included trials

First author	Year	Region	Type of study	Follow‐up	Participants	Female sex (%)	Treatment regimens
Wallentin^17^	2009	Asia and Australia, Europe, Middle East, America	multicenter	12 months	11 289	23.8	ticagrelor vs. clopidogrel
Tang^18^	2016	Asia	two‐center	6 months	400	28.0	ticagrelor vs. clopidogrel
Li^19^	2018	Asia	single‐center	12 months	442	21.7	ticagrelor vs. clopidogrel
Cai^20^	2015	Asia	single‐center	12 months	120	NA	ticagrelor vs. clopidogrel
Zeng^21^	2017	Asia	single‐center	12 months	204		ticagrelor vs. clopidogrel
Zhang^22^	2017	Asia	single‐center	6 months	181	49.0	ticagrelor vs. clopidogrel
Motovska^16^	2017	Czech Republic	multicenter	12 months	1230	NA	prasugre vs. ticagrelor
Patel^23^	2018	Asia	single‐center	12 months	1150	NA	prasugre vs. ticagrelor
Schüpke^14^	2019	Europe	multicenter	12 months	4018	23.8	prasugre vs. ticagrelor
Trenk^24^	2012	Europe and America	multicenter	6 months	423	27.4	prasugre vs. clopidogrel
Wiviott^8^	2007	Asia and Africa, Europe, Middle East, America	multicenter	15 months	13 608	26.0	prasugre vs. clopidogrel
Brener^25^	2014	Europe and America	multicenter	12 months	452	26.1	prasugre vs. clopidogrel
Montalescot^26^	2009	NA	multicenter	15 months	3534	22.6	prasugre vs. clopidogrel
Welsh^15^	2019	NA	multicenter	12 months	9932	23.8	prasugre vs. ticagrelor & ticagrelor vs. clopidogrel

### 3.2 Study quality

**FIGURE 2:**
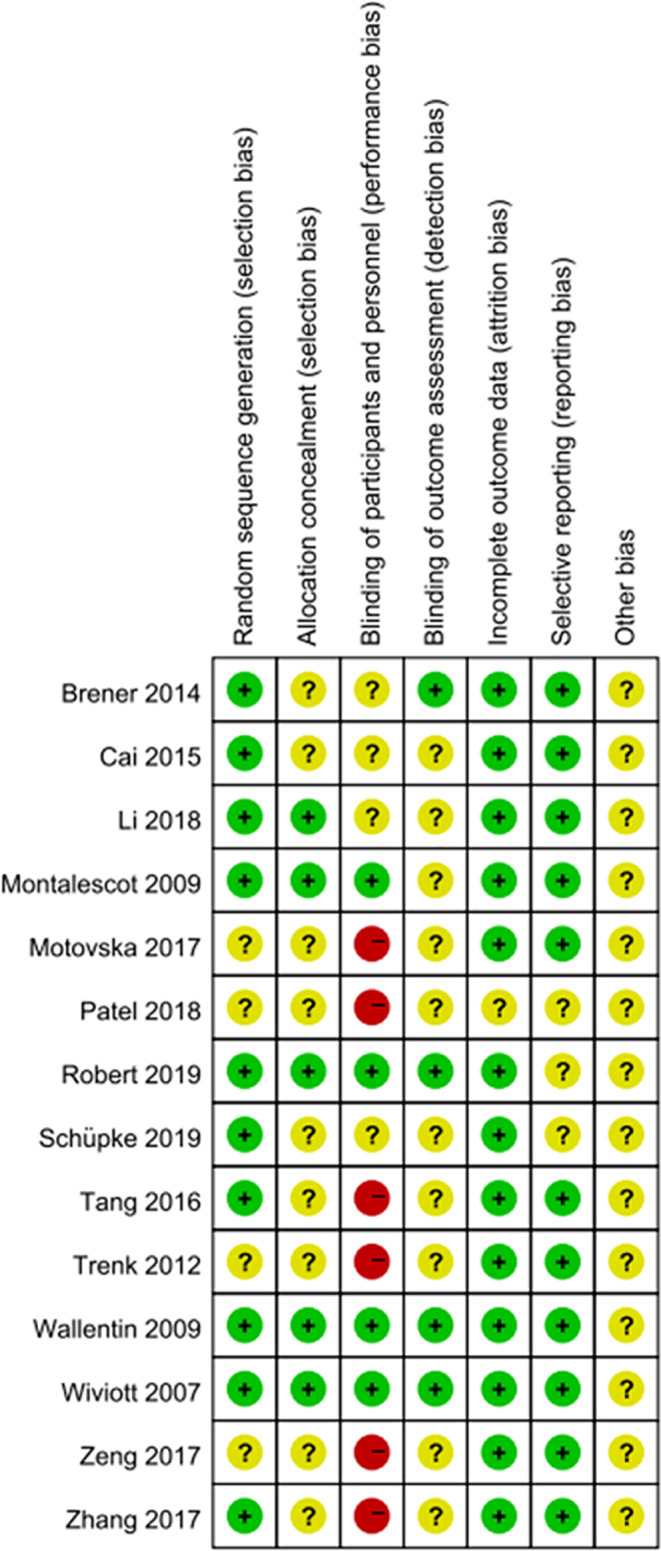
Risk of bias summary: the risk of bias of each domain in each study

**FIGURE 3:**
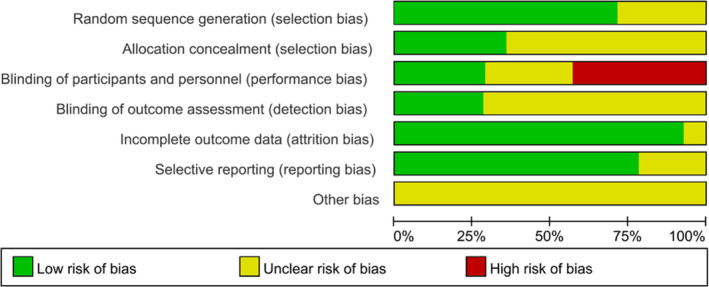
Risk of bias graph: an overall risk of bias of each domain. For example, the length of green rectangle means the number of studies being assessed as low risk of bias

The Cochrane Risk of Bias tool was applied for assessment of methodological quality.^27^ The quality evaluation of included studies was shown in Figures 2 and 3. Of the 14 studies included in this meta‐analysis, only four studies applied blinding method, while six studies had potential risk of bias due to lack of blinding for participants. In addition, the lack of clear statement of allocation concealment (in nine studies) and blinding of outcome assessment (in 10 studies) might result in potential risk of selection bias and detection bias, respectively.

### 3.3 Results of network meta‐analysis

**FIGURE 4:**
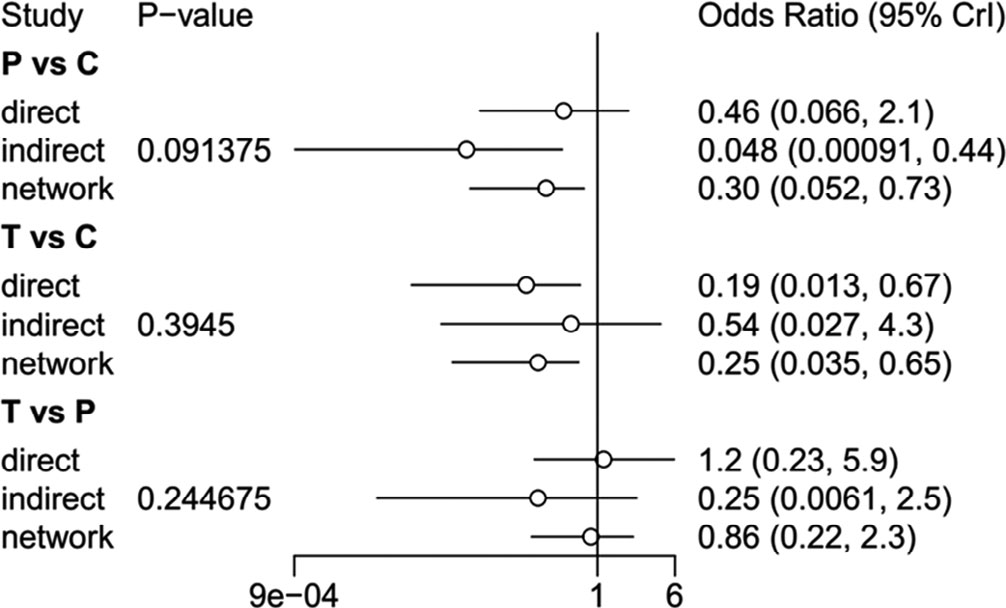
Network meta‐analysis results of ST among three DAPT regimen. C, clopidogrel; P, prasugrel; T, ticagrelor

As shown in Figure 4, a significant decrease in the frequency of ST was observed for prasugrel treatment compared with clopidogrel treatment (OR = 0.30, 95% CI = 0.052–0.73). Meanwhile, the frequency of ST was also significantly decreased for ticagrelor treatment compared with clopidogrel treatment (OR = 0.25, 95% CI = 0.035–0.65). No significant difference was observed between ticagrelor and prasugrel (OR = 0.86, 95% CI = 0.22–2.3).

### 3.4 Model convergence of our network meta‐analysis

The model convergence was checked using Brooks‐Gelman‐Rubin diagnostic statistics (Figure S1) and trace plots (Figure S2) for all model parameters. The Brooks‐Gelman‐Rubin statistics got close to one fast, which revealed that the three Markov chain Monte Carlo chains mixed well regardless of their different initial starting points. Meanwhile, the trace plots illustrated that every Markov chain Monte Carlo chains converged well.

### 3.5 Heterogeneity test and publication bias analysis

**FIGURE 5:**
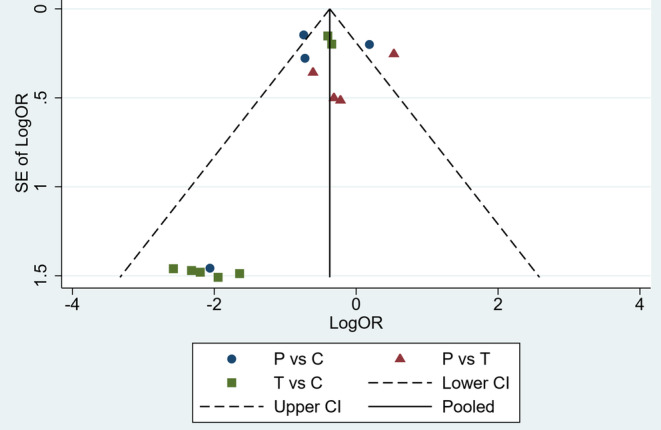
Funnel plot analysis on publication bias. P, prasugrel; T, ticagrelor; C, clopidogrel; OR, odds ratio; S.E. standard error; 95% CI, 95% confidence interval

No significant heterogeneity was observed across included studies (*P* = 0.980) (Figure S3). The identification of potential publication bias was estimated by funnel plot asymmetry (Figure 5). Moreover, Begg's test (*P* = 0.4) and Egger's test (t = −1.26, *P* = 0.229) showed that no publication bias was involved in this study.

## 4 DISCUSSION

Our network meta‐analysis indicated that prasugrel and ticagrelor had similar efficacy for preventing ST and both of them were more potent than clopidogrel. However, none of the three drugs could completely prevent ST.

As the most common complication, ST is a major clinical concern of post‐PCI treatment. Studies showed that the occurrence of ST was significantly related to inadequate antiplatelet therapy.^28^, ^29^ According to clinical guidelines, DAPT, with a combination of aspirin and one P2Y12 inhibitor (i.e., clopidogrel, prasugrel or ticagrelor), has been recommended to be used for antiplatelet therapy.^6^, ^30^ TRITON–TIMI 38 study revealed that prasugrel caused less ST than clopidogrel (68/6745 vs. 142/6653).^8^ Similarly, PLATO investigators reported that ticagrelor was more efficient on the prevention of ST events than clopidogrel (71/5569 vs. 106/5543).^17^ Our network meta‐analysis also obtained similar result. This might due to the different pharmacokinetics of these drugs. Prasugrel and ticagrelor generate active metabolite more rapidly than clopidogrel.^31^, ^32^, ^33^ The conversion of clopidogrel is linked to ABCB1 which act as a transporting molecule mediating the uptake of drug by intestinal cells, CYP2C19 and paraoxonase 1 (PON1) which affect the biotransformation of clopidogrel.^34^, ^35^, ^36^ This leads to a delayed anti‐platelet action of clopidogrel.^29^ By contrast, prasugrel requires only a single CYP‐dependent oxidative step during its metabolic transformation,^37^ while the action of ticagrelor needs no transformation.^32^, ^33^

Although ticagrelor has pharmacokinetic advantages over prasugrel, two indirect comparison meta‐analyses showed prasugrel might be more effective than ticagrelor for preventing ST.^12^, ^13^ However, subsequent head to head comparison studies, including the PRAGUE‐18 study (9/587 vs. 7/627), ISAR‐REACT 5 trial (22/1990 vs. 12/1994) and TOTAL trial (31/2188 vs. 30/1244), showed that patients treated with ticagrelor had a similar rate of ST compared to those treated with prasugrel.^14^, ^15^, ^16^ Consistently, our network meta‐analysis confirmed the similar efficacy for the prevention of ST between prasugrel and ticagrelor (OR = 0.86, 95% CI = 0.22–2.3).

More interestingly, several recent real‐world studies actually found that ticagrelor was not superior to clopidogrel for preventing ST,^38^, ^39^, ^40^ which was different from PLATO trial as well as our study. This might be explained by the following reasons. A potent mediator for efficacy attenuation of ticagrelor outside RCT might be decreased compliance because of the higher rate of adverse events (dyspnea, bleeding), administration of the drug twice daily and higher costs.^38^, ^39^, ^40^ Additionally, the overall improvement in the clinical outcomes of patients with ACS^41^ might be another possible explanation for the diminished benefit of ticagrelor in the modern era; particularly, this might be driven by progress in the use of drug‐eluting stents^42^ and poststenting care.

Finally, it should be noted that some patients still experience ST events in spite of DAPT. This might partly result from the inadequate anti‐platelet activity of DAPT. Besides, procedural “trauma” to the vessel and inadequate stent deployment play a role in the development of ST.^5^ Therefore, further research is needed to identify the mechanism of ST among individuals underwent PCI.

## 5 LIMITATIONS

Several limitations should be noticed in the present study. First, this study was a meta‐analysis of different trials, which were isolated designs. Although we included 46 983 patients in 14 studies, 34 829 (74.13%) were from three studies. These three studies weighted more in our pooled results than other 11 studies with small sample size. Therefore, it was apparent that the results in the three studies were more important due to not only large sample size but also high statistical power. Second, all the studies included in our network meta‐analysis are RCTs, and the conclusions may be discordant with real‐world studies because the RCT world does not correspond with the clinical practice. Therefore, this situation obviously limits the representability of our findings in current meta‐analysis. Third, different kinds of stents were used in the trials included in our study. Compared to bare‐metal stent, drug‐eluting stent was associated with a faster reendothelization and lower thrombotic risk. This might introduce bias in our results. Finally, due to lack of data, the effects of other high‐risk factors (i.e., type of myocardial infarction, complex coronary anatomy, and surgical factors) on ST were not evaluated in this study.

## 6 CONCLUSION

In conclusion, our findings suggest that prasugrel and ticagrelor are more effective than clopidogrel on prevention of ST among patients underwent PCI. Additionally, there is no significant difference in the prevention of ST between prasugrel and ticagrelor.

## CONFLICT OF INTEREST

The authors have no conflicts of interest to declare.

## AUTHOR CONTRIBUTIONS

Conceived and designed the meta‐analysis: Yuanmin Li and Haifeng Hou. Performed the study: Chen Zhang and Jian Zhao. Analyzed the data: Xiuxiu Xu and Qiang Xiao. Wrote the paper: Wenwen Chen.

## Supporting information


**FIGURE S1** Brooks‐Gelman‐Rubin diagnostic statistics. T, ticagrelor; C, clopidogrel; P, prasugrel; SE, standard error
**Figure S2** Trace plots of our models. T, ticagrelor; C, clopidogrel; P, prasugrel; SE, standard error
**Figure S3** Heterogeneity test. T, ticagrelor; C, clopidogrel; P, prasugrel; OR, odds ratio; 95% CI, 95% confidence interval

## Data Availability

The data supporting this network meta‐analysis are from previously reported studies and datasets, which have been cited.
