# Supplementary material for: Effects of clopidogrel, prasugrel and ticagrelor on prevention of stent thrombosis in patients underwent percutaneous coronary intervention: A network meta‐analysis
Source: Clin Cardiol. 2021 Mar 11;44(4):488–94. doi: 10.1002/clc.23536 (PMC8027588; doi:10.1002/clc.23536)
Supplement: Supplementary file 1 — FIGURE S1 Brooks‐Gelman‐Rubin diagnostic statistics. T, ticagrelor; C, clopidogrel; P, prasugrel; SE, standard error Figure S2 Trace plots of our models. T, ticagrelor; C, clopidogrel; P, prasugrel; SE, standard error Figure S3 Heterogeneity test. T, ticagrelor; C, clopidogrel; P, prasugrel; OR, odds ratio; 95% CI, 95% confidence interval [file CLC-44-488-s001.docx]

**[Supplementary materials]**

**
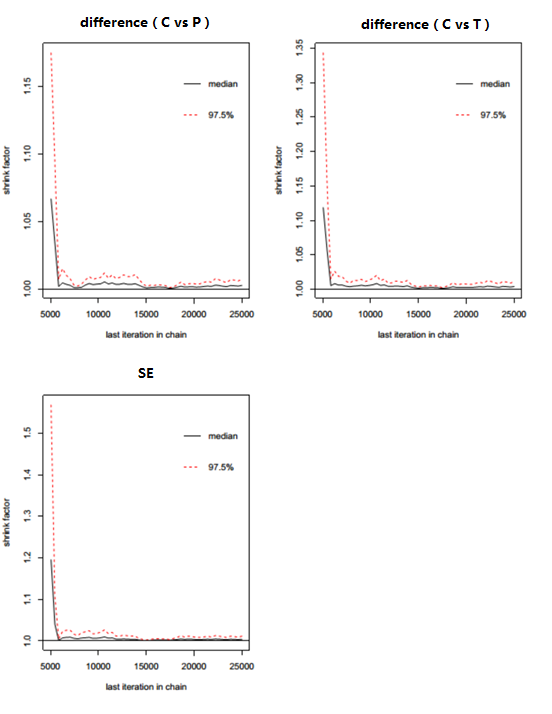
**

**FIGURE S1 Brooks-Gelman-Rubin diagnostic statistics**

T, ticagrelor; C, clopidogrel; P, prasugrel; SE, standard error

**
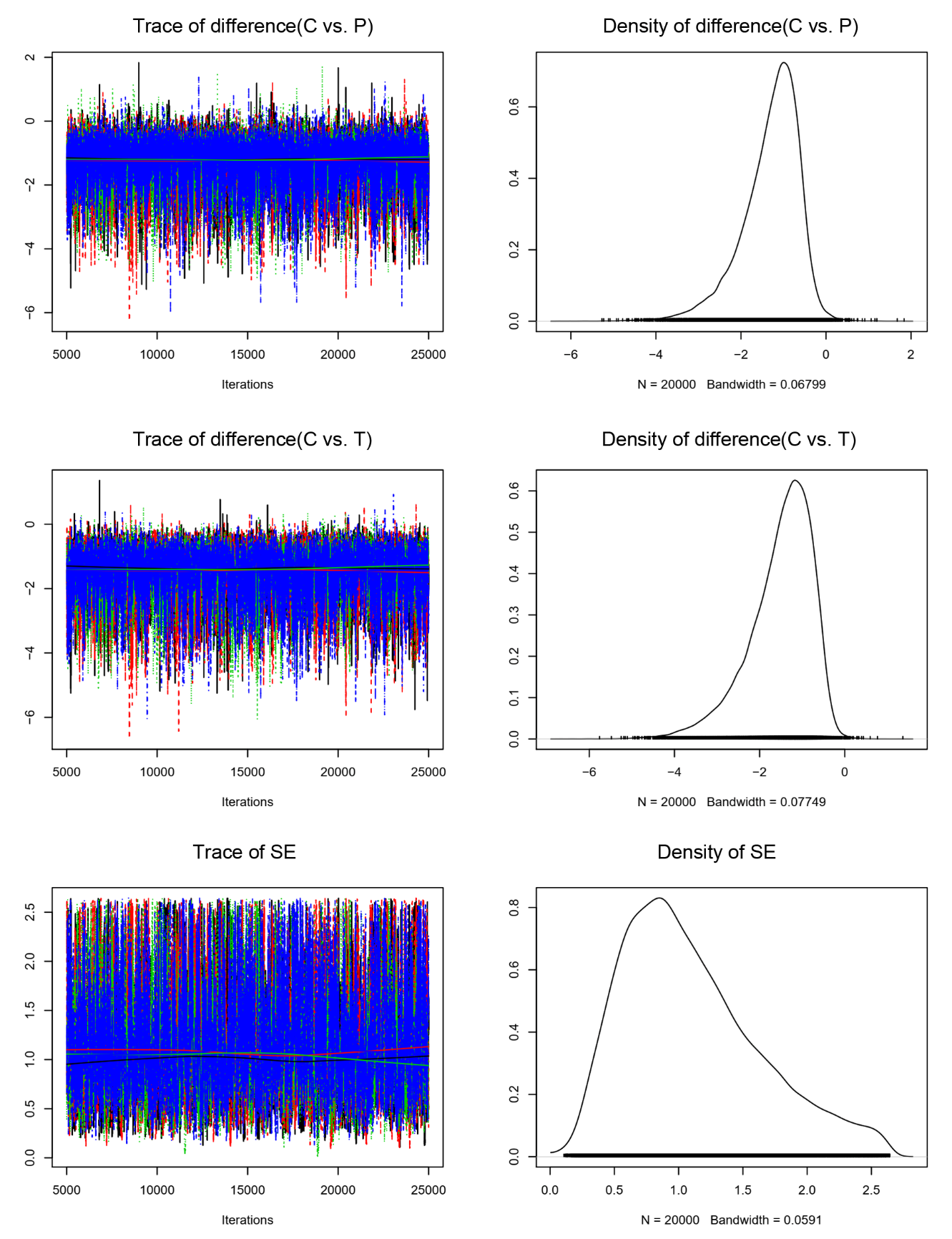
**

**FIGURE S2 Trace plots of our models**

T, ticagrelor; C, clopidogrel; P, prasugrel; SE, standard error

**
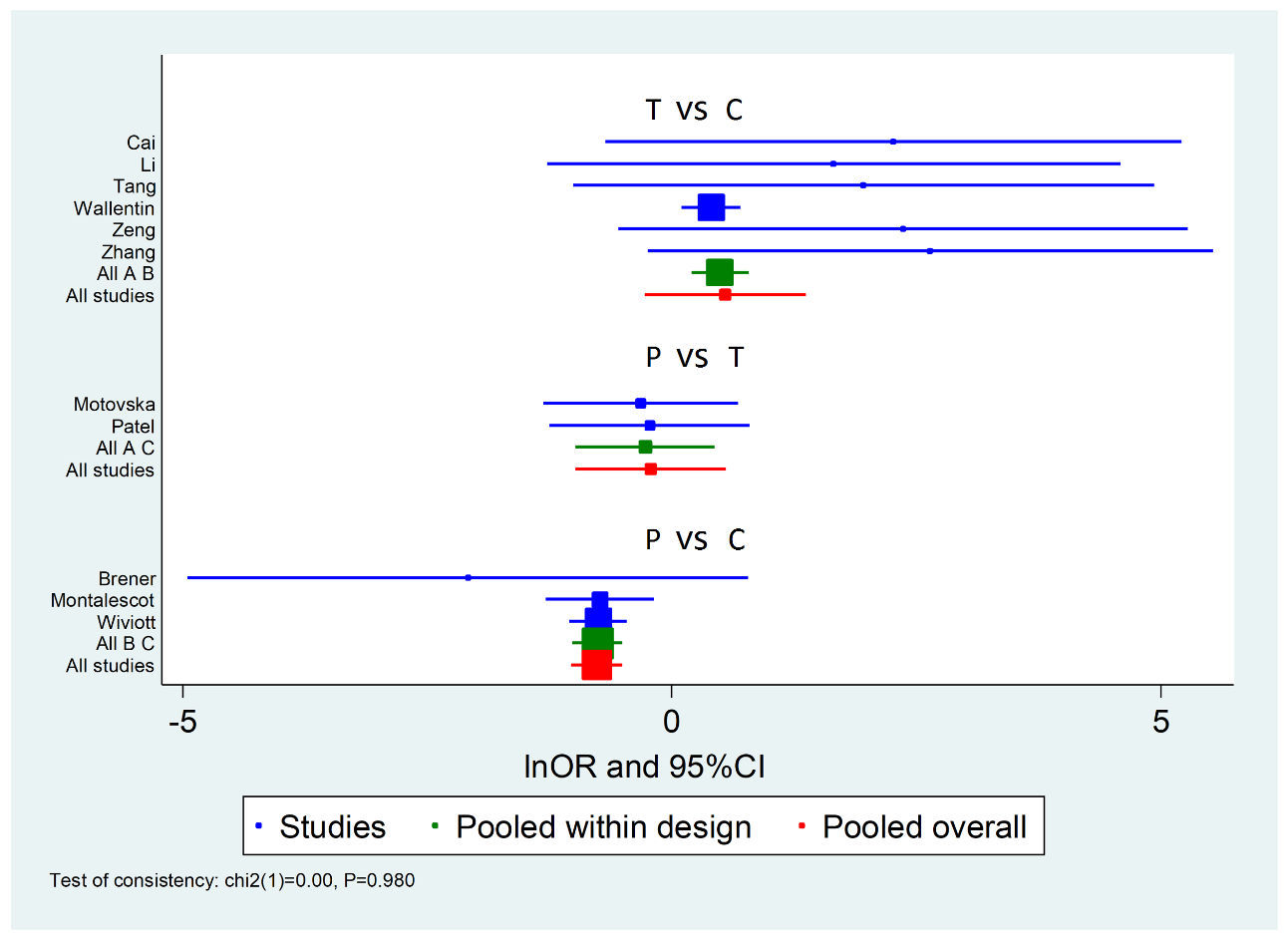
**

**FIGURE S3 Heterogeneity test**

T, ticagrelor; C, clopidogrel; P, prasugrel; OR, odds ratio; 95%CI, 95%confidence interval
